# Low-cost optical sensors in electrified lab-on-a-disc platforms: liquid-phase boundary detection and automated diagnostics

**DOI:** 10.1038/s41378-025-00896-5

**Published:** 2025-04-07

**Authors:** Vahid Kordzadeh-Kermani, Maryam Vahid, Seyed Nezameddin Ashrafizadeh, Sergio O. Martinez-Chapa, Marc J. Madou, Masoud Madadelahi

**Affiliations:** 1 School of Engineering and Sciences, Tecnologico de Monterrey, Monterrey, NL Mexico; 2 Research Lab for Advanced Separation Processes, Department of Chemical Engineering, Iran University of Science and Technology, Narmak, Tehran, Iran; 3 Department of Mechanical Engineering, Isfahan University of Technology, Isfahan, Iran; 4 Department of Mechanical and Aerospace Engineering, University of California Irvine, Irvine, CA USA

**Keywords:** Microfluidics, Biosensors

## Abstract

Centrifugal microfluidic platforms are highly regarded for their potential in multiplexing and automation, as well as their wide range of applications, especially in separating blood plasma and manipulating two-phase flows. However, the need to use stroboscopes or high-speed cameras for monitoring these tasks hinders the extensive use of these platforms in research and commercial settings. In this study, we introduce an innovative and cost-effective strategy for using an array of light-dependent resistors (LDRs) as optical sensors in microfluidic devices, particularly centrifugal platforms. While LDRs are attractive for their potential use as photodetectors, their bulky size frequently restricts their ability to provide high-resolution detection in microfluidic systems. Here, we use specific waveguides to direct light beams from narrow apertures onto the surface of LDRs. We integrated these LDRs into electrified Lab-on-a-Disc (eLOD) devices, with wireless connectivity to smartphones and laptops. This enables many applications, such as droplet/particle counting and velocity measurement, concentration analysis, fluidic interface detection in multiphase flows, real-time monitoring of sample volume on centrifugal platforms, and detection of blood plasma separation as an alternative to costly stroboscope devices, microscopes, and high-speed imaging. We used numerical simulations to evaluate various fluids and scenarios, which include rotation speeds of up to 50 rad/s and a range of droplet sizes. For the testbed, we used the developed eLOD device to analyze red blood cell (RBC) deformability and improve the automated detection of sickle cell anemia by monitoring differences in RBC deformability during centrifugation using the sensors’ signals. In addition to sickle cell anemia, this device has the potential to facilitate low-cost automated detection of other medical conditions characterized by altered RBC deformability, such as thalassemia, malaria, and diabetes.

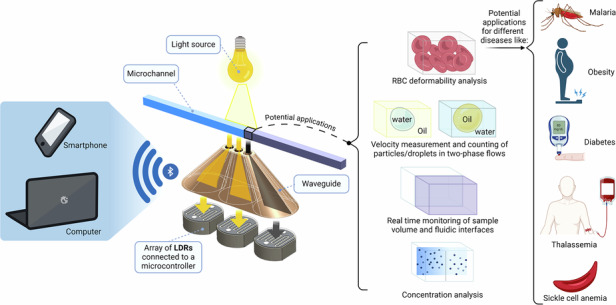

## Introduction

In recent years, microfluidic devices have been extensively used as reliable tools for a wide range of applications, from micro- and nanomaterial synthesis^1–3^, to medical diagnostics^4,5^. Depending on the predetermined and pre-designed objectives of the microfluidic devices, they may need to be equipped with multiple signal readout options for proper operation and data extraction. Hence, observing reproducible analytical signals from state-of-the-art microfluidic devices is required and known as a challenging issue that guarantees the reliable performance of lab-on-a-chip (LOC) platforms^6,7^. To date, various signal acquisition strategies have been integrated into LOCs, such as calorimetry/temperature monitoring^8^, light/color sensing^9^, electrical/magnetic properties monitoring, *etc*^10^. Among these methods, measuring the light intensity and color variations provided straightforward mechanisms for accurately detecting and assaying analytes in LOCs^11^

The light/color-based signal readout techniques (optical methods) can be implemented into LOCs using different components, including light sources (e.g., laser rays, visible, infrared, or ultraviolet rays at a wide range of wavelengths)^12^, detectors (e.g., light intensity sensors, red/green/blue (RGB) sensors, spectrophotometers, *etc*.)^13^, and mirrors in both on-chip and off-chip configurations^14^. These techniques provide highly sensitive and reproducible signals, especially for detecting low-concentration analytes, which make them suitable for use in point-of-care (POC) microfluidic devices ^15,16^.

Generally, optical detection methods require costly, bulky modules and parts, which limit their application in microfluidic devices. Accordingly, researchers have been very interested in developing cost-effective and optimized optical detection methods in recent years. In this regard, the combination of light-emitting diodes (LEDs) (as light sources) and light-dependent resistors (LDRs) (as detectors) in a micro-controlled environment has been proposed for measuring the light absorbance in LOCs as a cost-effective and sensitive signal readout strategy for small sample volumes. Commercial LDRs are low-cost electronic parts that are often available in a (semi) circular shape (diameter ≈5 mm) with cadmium sulfide (CdS) as the photoconductive material inside. When the light intensity increases toward an LDR (with a response time of 8 to 12 ms), the electrical resistance decreases, and vice versa^17^. Therefore, by exploiting this inherent characteristic of LDRs, a wide range of applications can be defined in microsystems by monitoring their electrical resistance.

So far, a single LDR-LED pair has been used in some different microfluidic devices. Detecting chemical substances and diagnosing diseases in microfluidic devices are the primary objectives of the associated studies that utilized the LDR–LED pair. Consequently, scientists have developed devices that use an LED-LDR pair positioned opposite each other to identify various chemical substances (e.g., phosphate, ammonium^18^, and glutathione^19^) based on their color-dependent concentrations. These studies also include medical pocket-sized POC devices, such as those for detecting *β*-hydroxybutyrate (a marker of liver performance in oxidizing fatty acids)^20^ and *β*-2-microglobulin in human tear samples (an indicator of diabetes mellitus)^21^. Cell viability analyses for MCF7 species were also performed using the LDR photodetection method, which monitored the concentration of hydrogen peroxide excreted by cells^22^. Additionally, some studies used single LDR-LED pair in two-phase flow within capillary tubes to measure the number, size, velocity, or flow rate. However, due to the use of only one LDR, its large size, and the lack of modifications like waveguides, their analysis was limited to large-sized and stretched fluidic slugs, comparable to or much larger than the LDR^23,24^. In this way, Coliaie et al. proposed a device for detecting liquid-liquid phase (oil/water emulsion) boundaries in a flowing fluid used in pharmaceutical drug synthesis procedures ^25^.

Centrifugal microfluidics, also known as Lab-on-a-Disc (LOD) or lab-on-a-CD, is a particular subcategory of microfluidic platforms that exploit centrifugal forces to move fluids in microchannel networks^16^. These devices attracted considerable attention due to their simplicity of implementation, lack of need for external pumps and tubing, and ability to multiplex various processes, making them suitable for automated POC applications^26,27^. Electrified LOD (eLOD) has emerged in recent years, allowing for the integration of various electricity-required functions such as on-disc sensors and electronic modules. Therefore, several signal readout strategies can be implemented on eLODs for multiple purposes^28^. Various photo/electrical detection approaches have been implemented on eLODs based on on-disc and off-disc configurations^29^. Off-disc and on-disc imaging were among the primary requests of researchers working with centrifugal microfluidics. As first attempts, Kazarine and Salin prepared off-disc capturing setups that monitored the aliquoting process of chambers during rotation under strobe illumination (up to 1400 rpm)^30^. In the following, Serioli et al. developed a wireless eLOD equipped with a co-rotating on-disc camera to analyze the mixing of two colorful fluids under different rotation speeds^31^. Delgado et al. have devoted considerable effort to developing signal acquisition methods for eLODs^32^. They integrated a silicon-based photomultiplier device to detect human C-reactive protein in a chemiluminescent scenario using a wireless eLOD^33^. Their results were reported to be reasonable in comparison with commercial analyzers. They also developed another eLOD that was utilized in various colorimetry, thermometry, and electrochemical measuring processes by exploiting RGB, infrared (IR), and potentiostat sensors, respectively^34^. Electrochemical sensing is another signal-sensing method that has been integrated into eLODs. Accordingly, Abi-Samra et al. measured fluid velocity by placing interdigitated electrodes inside a microchannel connected to an off-disc potentiostat^35^. Their results showed suitable compliance with other velocimetry methods up to 1000 rpm rotation speed.

**Fig. 1:**
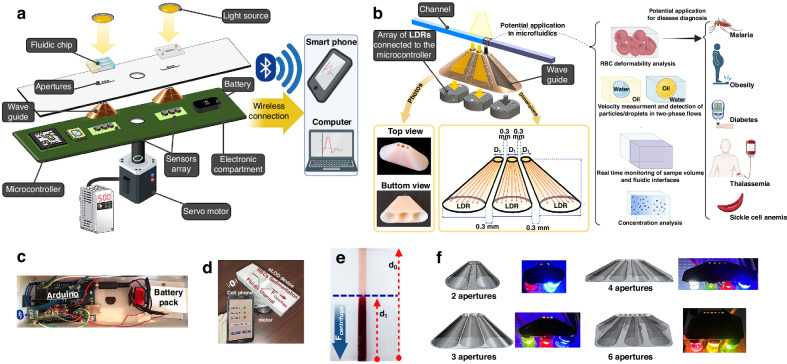
The designs, photographs, and detection process in the proposed method. **a** A schematic illustration of the eLOD device mounted on a rotor, including servo motor and drive, electronic compartment (containing microcontroller, arrays of three adjacent LDRs, and battery pack), cone-shaped waveguides, fluidic chips, and light sources. The microcontroller is incorporated with a Bluetooth® module, providing wireless connectivity to smartphones and laptops. Two arrays of photodetectors are radially positioned beneath the fluidic channel. **b** The illustration shows the mechanism of the waveguides. A colorful fluid (designated by violet) in the fluidic channel is placed over one of the apertures, hindering the light and decreasing the intensity that reaches the LDR. As the colorful fluid moves, it affects more photosensors. The cone-shaped waveguides act as transmitters, directing light from a narrow aperture to the LDR surface. The geometric details of the cone-shaped waveguides (*D*_1_ = 1 mm, the aperture diameter, *D*_2_ = 5 mm, the base diameter matching LDRs, with a 0.3 mm gap between cones), with top and bottom photos of a waveguide (other *D*_1_ sizes have also been investigated). By exploiting this light detection mechanism at sub-millimeter distances, various color-based applications are enabled, such as measuring RBC deformability in bulk form, measuring droplet/particle velocity in two-phase flows, monitoring sample volume, and detecting concentration inside microfluidic devices. Different associated diseases can be diagnosed by measuring the deformability of RBCs (e.g., Malaria, Obesity, Diabetes, Thalassemia, and Sickle cell anemia). **c** The photo of the internal view of the electronic compartment, including LDRs, Arduino controller, Bluetooth® module, and battery pack. **d** The photo of the assembled eLOD device and the fluidic chips on the motor, connected via Bluetooth® to a smartphone (apertures array located beneath the fluidic channels). **e** Illustration depicting RBC sedimentation height in the fluidic channel during centrifugation (1600 rpm) on the eLOD device, where *d*_0_ is the fluidic channel length, and d_1_ is the sedimentation height of RBCs post-centrifugation. **f** 3D illustration of various waveguide structures with different numbers of apertures, ranging from 2 to 6. The photographs next to the 3D designs demonstrate the transmission of colorful light from LED sources positioned beneath the cone-shaped waveguides. The light is conveyed from the base of the cone to the aperture openings (*D*1 = 1 mm), showcasing their light-guiding capability

In light of the information provided above about photodetectors used in LOC/LOD platforms, a low-cost, high-resolution, and user-friendly photodetection method in microfluidic devices, particularly in eLODs, is highly valued. In this study, we present a method for utilizing an array of LDRs to enhance their planar resolution for detecting light intensity variations and boundaries in two-phase flows. To address the challenges posed by the large size of commercial LDRs, as shown in Fig. 1, we utilized an array of LDR sensors equipped with waveguides. This configuration was subsequently integrated into an eLOD platform, as illustrated in Fig. 1a. This platform contains electronic parts, cone-shaped waveguides, and fluidic chips. The waveguide sensors are designed to be reusable and can be utilized for multiple cycles and applications, ensuring proper alignment beneath the sensing area. In Fig. 1b, the mechanism incorporated with this measurement approach is shown. By placing a colorful fluid over one of the narrow apertures at the top of the waveguides, the fluid absorbs some light, reducing the intensity that reaches and is detected by the LDRs by casting a shadow. By moving the colorful fluid (or droplet and particle), the cast shadow will affect more apertures, enabling distance-dependent detections. These waveguides act as transmitters for conducting the light rays from a small aperture at the top of the cones (*D*_1_ = 0.2, 0.4, 0.6, 0.8, and 1 mm) to the bottom, where the LDRs are positioned (*D*_2_ = 5 mm). This approach enables precise photodetection on eLODs/LOCs at sub-millimeter intervals, enabling various color-dependent applications, including measuring the RBCs deformability in bulk form, measuring the velocity of droplet/particle in two-phase flows, monitoring the volume of samples, and detecting the concentration inside the microfluidic devices. In this way, different associated diseases can be diagnosed by measuring the deformability of RBCs (e.g., Malaria, Obesity, Diabetes, Thalassemia, and Sickle cell anemia). After the calibration of sensors, we assessed the efficiency of this device for several applications (based on the color difference of two fluids): (1) detection and counting of water-in-oil and oil-in-water dyed droplets; (2) detecting the interface of two-phase flows and sample volume measurement; (3) measuring the separation time of RBCs and plasma in a whole blood sample during centrifugation; (4) measuring the deformability of healthy and hardened RBCs under centrifugation (in a bulk form); and (5) detecting sickle cell anemia disease based on the deformability of RBCs. Additionally, we conducted a numerical simulation to extend and predict the system’s behavior at higher rotation speeds and with various fluid types. Our eLOD device is capable of signal processing with an integrated microcontroller and can wirelessly connect to smartphones and laptops via Bluetooth^®^ for further post-processing. This newly proposed, cost-effective optical detection method, which allows for varying the number and arrangement of LDRs, is applicable not only to eLODs but also to non-centrifugal LOC systems across various applications.

## Results and discussion

**Fig. 2:**
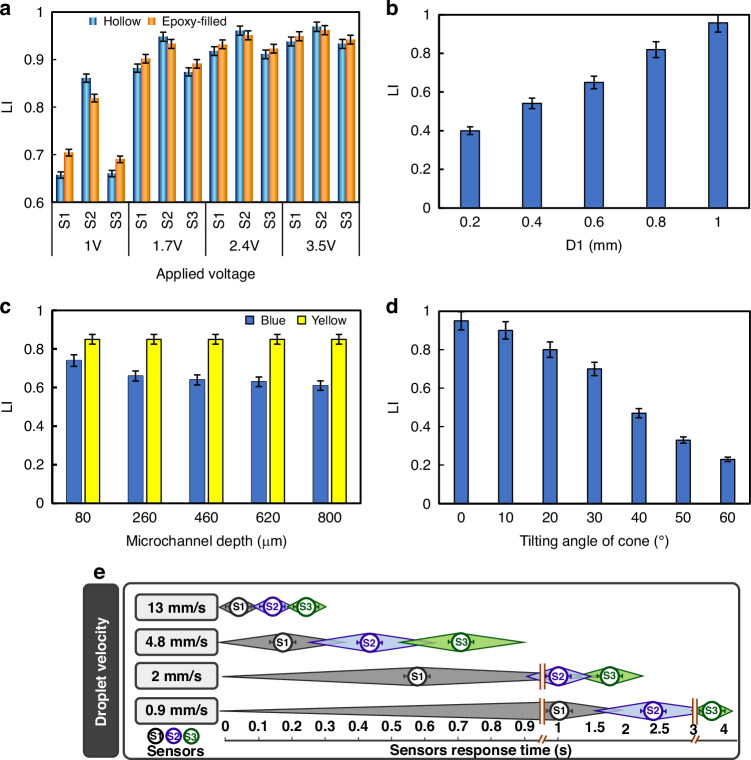
Characterization of light sensors array in different conditions. Results of the measured LI parameters using cone-shaped waveguide for **a** different light powers (LED voltages) for hollow and filled cone-shaped waveguides, **b** different aperture sizes, **c** microchannel depth for two colors of blue- and yellow-colored aqueous fluids (100% concentration; see method section), and **d** cone’s tilting angle. Results are reported as the average with a 5% deviation. **e** The sensors’ (S1, S2, and S3) response time at different velocities of a 1-mm diameter transparent water droplet in blue dyed mineral oil as continuous phase (13, 4.8, 2, and 0.9 mm/s at 240, 170, 95, and 64 rpm rotation of eLOD, respectively. *D*_1_ = 1 mm). The droplet velocities indicated on the left side of the figure were determined by examining video frames taken with an on-disc microscope. These values matched the velocities calculated using the photodetectors’ signals by dividing the distance between the apertures by the elapsed time. The vertical size of the triangular shapes at each time indicates the light intensities that reach each LDR, showing the passage of the droplet over the sensors’ position, which gradually increases the detected light intensity by covering the entire sensing area (see Video S1). Black circles, including S1, S2, and S3 inside, indicate the time at which the droplets coincide with the sensors’ position. In all the sections, *D*_1_ = 1 mm, the number of apertures in the waveguide is 3, and the LED power is 3 V unless otherwise stated. Error bars represent standard deviations

As previously noted, increasing the light intensity results in decreased electrical resistance in LDRs. To explore this correlation, we conducted experiments with varying light intensities by adjusting LED voltage directed at the array of three sensors. The waveguides were categorized into two categories: hollow and epoxy-filled. Sensor output signals were normalized to a scale of 0 to 1. This normalization was achieved by determining the minimum and maximum electrical resistances exhibited by the LDRs. In this normalized scale, 0 corresponds to absolute darkness, while 1 corresponds to the highest level of light illumination achievable by using the given LED power. Figure 2 illustrates outcomes using the light intensity (LI) parameter, reflecting the proportion of light rays detected by three LDR sensors. For example, an LI value of 0.6 means that the LDR detected 60% of the light rays at the given LED power, while the remaining 40% were blocked or absorbed by a colorful fluid. An LI value of 1 indicates a situation where there is no colored solution covering the sensor, allowing the detection of the highest level of light intensity using the cone-shaped waveguides.

In Fig. 2a, we show the impact of varying the LED power on the electrical resistance (or LI) of sensors 1, 2, and 3. Sensors 1 and 3 correspond to the two oblique cones, while sensor 2 is the middle vertical cone (see Fig. 1b). Under absolute darkness, the LDR has the highest electrical resistance (approximately corresponding to an LI of 0.02). As the voltage applied to the LED increases (resulting in more powerful light) up to 3.5 V, the LI value rises to about 0.95. Interestingly, the epoxy-filled oblique cones (sensors 1 and 3) displayed superior light-guiding capabilities towards the LDRs compared to the hollow cones. This enhanced performance can be attributed to the cloudy, solid medium provided by the cured epoxy resin, which possesses a higher light refractive index (≈1.5)^36^. This characteristic led to improved scattering of light rays along indirect paths within the oblique cones. The presence of cured epoxy resin in the vertical cone (sensor 2) caused a slight decrease in LI due to its absorption of light in the direct pathway of light rays. Consequently, filling the cones with epoxy resin enhances the re-emission of light rays within the oblique cones while slightly impeding the transmission of light rays through the central vertical cone. At lower LED power, the oblique epoxy-filled cones demonstrate up to 5% improvement in light transmission compared to the hollow cones. Since the waveguide is designed to perform effectively under varying light conditions, from low light with small aperture sizes (e.g., 0.2 mm) to larger apertures, we opted to proceed with experiments using three epoxy-filled cones.

Figure 2b illustrates how changes in aperture diameter (*D*_1_) affect the amount of light reaching the sensors, ranging from 0.2 to 1 mm at a 3.5 V LED voltage (Fig. S1 shows the photos of pristine waveguides with different aperture sizes). As the aperture diameter of the waveguides increases, more light reaches the sensors. Notably, even with small apertures (*D*_1_ = 200 µm), the cone-modified sensors effectively detect light, indicating their suitability for highly sensitive sub-millimeter detection applications. In Fig. 2c, the impact of the microchannel depth on the LI parameter is shown for various depths ranging from 80 to 800 µm. The experiment utilized blue- and yellow-dyed aqueous solutions at 100% concentration (see the Methods section). Hence, increasing liquid depths simulates higher dye concentrations, decreasing the LI parameter for blue-dyed solutions from 0.74 to 0.6 as the depth increases. Conversely, a slight change is noted for the LI parameter in the case of yellow-dyed solutions. Based on this observation, it is suggested that this method is also applicable to thin microchannels, where it generates signals based on color intensity differences, enabling the differentiation of multiple fluids or droplets by imposing varying light intensity values toward LDRs. Figure S2a, b illustrates the effect of dye concentration on light absorbance for oil-based and water-based colored liquids, respectively. It was observed that an increase in color concentration led to a decrease in the LI value. This decrease in LI of these different concentration liquids occurs due to the darkness caused by the higher dye concentrations acting as a light barrier, similar to observations in Fig. 2c for liquid depth. Adjustments in yellow color concentration in water solution (as shown in Fig. S2b) have minimal impact on the LI parameter (approximately 2% variation). This minimal effect is attributed to the transparent nature of yellow dye, which allows light rays to transmit towards the sensor. Similarly, trends in the color density of oil samples reflect up to 40% decrease in the LI parameter with an increase in dyed oil concentration (for brown color as demonstrated in Fig. S2). Therefore, this sensor array can detect and differentiate the light intensity based on the color of aqueous and oil fluids or on the concentration of analytes that influence the sample’s transparency or color. The effect of the cone tilting angle is shown in Fig. 2d (See Fig. S2c). It is demonstrated that as the tilt angle of the cone increases, the detected light intensity decreases due to the increased distance between the aperture and the LDR surface that the light must pass through.

Figure 2e shows how sensors (S1, S2, and S3) respond to different speeds of a 1-mm transparent water droplet moving through a dyed oil-continuous phase (See Video S1). This figure demonstrates that this array of sensors can detect droplet movement at velocities up to 13 mm/s by providing at least a 0.1 s interval between each sensor response time. The detection of higher velocities is achievable using this configuration of miniaturized light sensors due to the fast response time of the LDRs (≈10 ms). However, in our setup, the measurement of higher droplet velocities was unattainable due to the viscosity of the oil phase and the limited cross-section area of the channel, which restrict the maximum velocity of the fluid/droplet at the selected rotation speeds. In Fig. 2e, the vertical size of the triangular shapes at each time indicates the light intensity that reaches the related sensor. During the passage of the droplet through the channel, the detected light intensity gradually increases until it coincides with the aperture position. By moving the droplet over the sensor’s position, the light intensity decreases again. As the photosensors had a 0.3 mm gap, a 1 mm droplet covered some parts of two adjacent sensors simultaneously during its movement. Video S2 illustrates the opposite scenario: the movement and velocity of a dyed water droplet in transparent oil at different rotation speeds. Here, the signal behavior is reversed, with the dyed droplet blocking the light as it approaches the sensors, resulting in a decrease in LI. In the following sections, we explore different applications of this optical detection approach using the array of sensors: experimental optical detections (including droplet sensing and counting, two-phase interface detection, sample volume detection, and diagnostic applications), as well as numerical simulations.

### Experimental optical detections

#### Detection and counting of water-in-oil and oil-in-water droplets

**Fig. 3:**
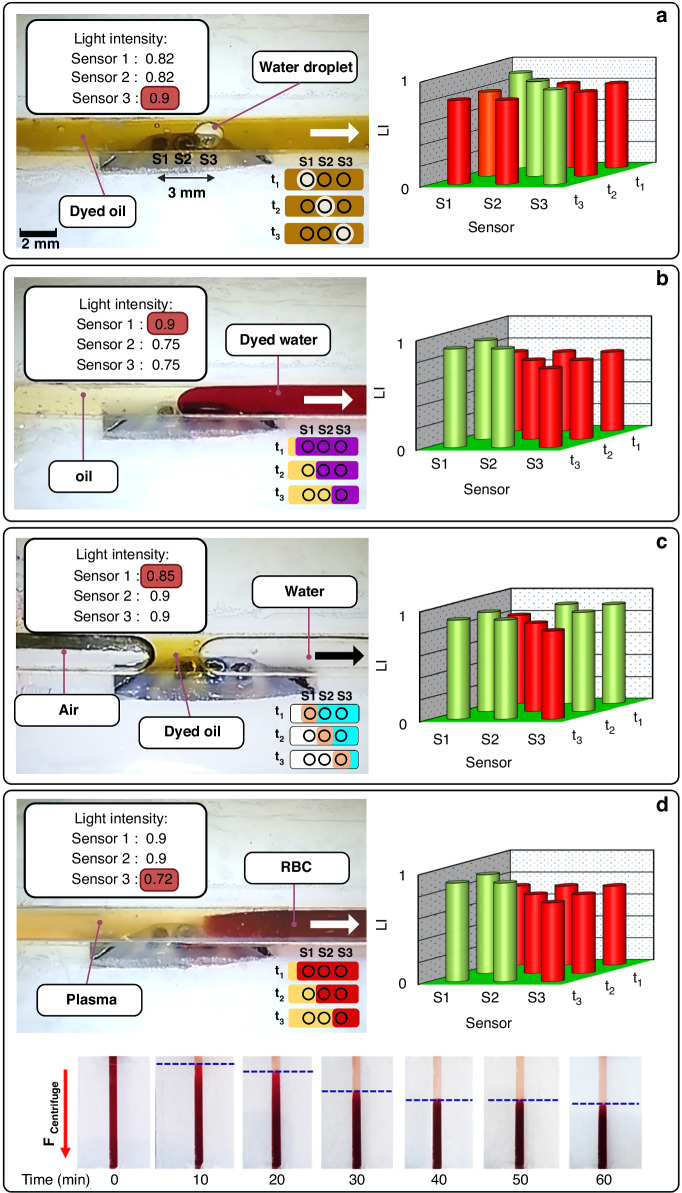
The photographs of sensors, various types of colorful fluids, and their corresponding responses. **a** Detection of water droplets in a dyed-oil continuous phase. **b** Detection of a two-phase flow interface based on the color difference between the aqueous and oil phases. **c** Detection of a varying sample volume in the channel. **d** Plasma-RBC separation in a whole blood sample within the channel, with sensor array response. The bottom image shows the plasma-RBC separation process during eLOD centrifugation (1600 rpm, up to 60 min), captured by a photograph and used as a reference for evaluating sensor array performance. In the 3D bar charts, green columns represent activated sensors (receiving a higher amount of light intensity), while red columns represent deactivated sensors (receiving a lower amount of light intensity). A minimum 5% difference in LI absorbance was used for detection. In the images, any sensor value number deviating from the other two is highlighted in red. The arrows indicate the direction of fluid flow (scale bar: 2 mm). *t*_1_, *t*_2_, and *t*_3_ represent the times at which the colored fluid or droplet was positioned over the corresponding sensor

Figure 3a shows the detection of a water droplet in the dyed oil. As the droplet moves, the color variation and the associated shadow create varying light intensities, detectable by the array of sensors. Sensors 1, 2, and 3 responded at the times of *t*_1_, *t*_2_, and *t*_3_, respectively, determined the droplet’s position. As an example shown in the image, a large droplet exceeding the sensor aperture (*D*_1_ = 1 mm) yields an LI parameter of approximately 0.9. Smaller droplets also change the LI as they move over the sensor apertures. This setup successfully detected droplets as small as 200 µm by reducing the waveguide aperture size (see Fig. S3), as discussed regarding aperture diameter in Fig. 2b. This sensor configuration is also suitable for various similar applications, including droplet counting, light intensity, and size-based matter/structure sorting, and speed measurement as they traverse the sensor apertures. All these capabilities can be incorporated into eLOD and LOC devices.

### Real-time sample volume monitoring and fluidic interface detection

In Fig. 3b, we illustrate the detection of the interface (boundaries) between two immiscible fluids. Changes in the fluid boundaries within the microchannel alter the sensor response as the color shifts at different times t_1_, t_2_, and t_3_, resulting in different LI parameters from 0.75 to 0.9. Furthermore, real-time monitoring of sample volume is depicted in Fig. 3c. In this case, a small volume of dyed oil acts as an indicator, moving over the sensors according to the volume of the transparent sample in the channel. This indicator covers the sensor aperture at different times and positions. This application is beneficial in centrifugal microfluidics for accurately monitoring the sample volume at high rotational speeds. By detecting the interface and knowing the cross-sectional area of the microfluidic chamber or channel, the volume can be accurately calculated in real time.

#### Diagnostics

We used our eLOD device to centrifuge a whole blood sample, capturing the separation of plasma and RBCs’ sedimentation at 10-min intervals at 1600 rpm (Fig. 3d). Similar to the two-phase detection method mentioned earlier, the difference in color and light absorbance of plasma and RBCs results in different responses from sensors 1, 2, and 3 during centrifugation. This observation enables precise tracking of the plasma or RBC fractionation when it covers the respective sensors, making the detection of boundaries at different time intervals possible. In comparison to traditional methods for LOD devices, like utilizing a stroboscope, our array of sensors equipped with cone-shaped waveguides provides a cost-effective, precise, and real-time solution. It enables tracking of the RBC-plasma interface at high rotational speeds. (While a stroboscope typically costs around $500, the total expense of our eLOD—including Arduino, modules, and sensors—is under $50). The contrast in the LI parameter between the plasma-separated fraction (0.9) and the RBC-separated fraction (0.72) is demonstrated in Fig. 3d. This notable difference in light absorbance presents significant potential for various applications in blood chemistry. The variation in LI between plasma and RBC can serve as a criterion for automated assessment of the deformability of RBCs, a sedimentation-related property of bulk samples containing RBC. Deformable and non-deformable RBCs exhibit different sedimentation heights^37^, facilitating detection through measurements of the sedimentation index (SI) parameter at specific time intervals by receiving signals from the linear array of sensors.

**Fig. 4:**
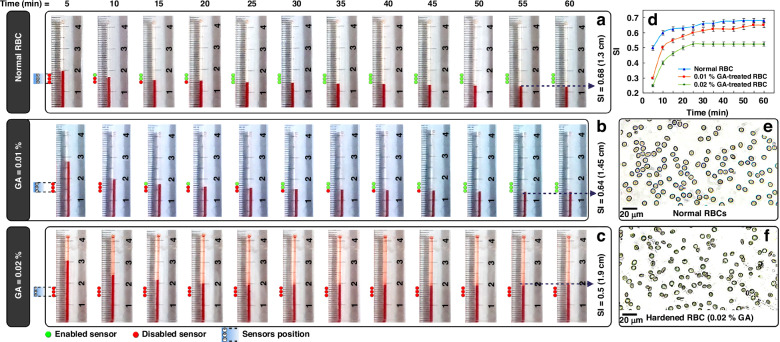
The photos of the fluidic channel, including different hardened blood samples during centrifugation, the response of the sensors, and the microscope photographs of blood samples. Measuring sedimentation height and ultimate sedimentation index (SI) for different RBC samples at various time steps: **a** normal RBCs (SI = 0.68); hardened with GA at concentrations of (**b**) 0.01% (SI = 0.64) and **c** 0.02% (SI = 0.5). Red and green circles show sensor responses. Red-filled circles indicate sensors covered by the RBC fraction during centrifugation, casting shadows over sensor apertures and reducing detected light intensity. Green-filled circles indicate sensors covered by PBS-separated fractions, allowing more light intensity to reach the sensors. In all experiments, the sensor array was placed at a height of 14–18 mm. Photos of blood sedimentation were captured under centrifugation using a synchronized strobe light at 5-min intervals. Channels were fully filled with RBC samples, maintaining consistent initial heights across experiments. Each sample showed a different number of enabled sensors after 15 min of centrifugation (normal RBCs: 2 sensors, 0.01% GA-treated RBCs: 1 sensor, 0.02% GA-treated RBCs: no sensor, *D*_1_ = 1 mm, LED applied voltage = 3.5 V). This method enables differentiation of deformability among differently treated RBC samples by counting enabled sensors. **d** The graph shows sedimentation indices for different RBC types, normal and hardened, during 60 min of centrifugation (Error bars represent standard deviations). The values were derived from the recorded video as a reference for evaluating sensor array performance. **e** Microscope images of normal RBCs and **f** hardened RBCs with 0.02% GA

A variety of diseases, such as sickle cell anemia, thalassemia^38^, malaria^39^, and diabetes^40^, can impact RBC deformability^41,42^. Therefore, it is crucial to quantify RBC deformability and utilize it alongside other diagnostic techniques. Various methods have been introduced to measure the deformability of RBCs^43^. The deformability of RBCs in this study has been analyzed in bulk form but not using a single-cell approach. In this regard, when a sample including RBCs has high deformability, it generates lower sedimentation heights, representing the inherent ability of RBCs to form a denser aggregate volume. The less deformable RBCs generate higher sedimentation heights than the highly deformable RBCs. Accordingly, our array of sensors, capable of detecting with sub-millimeter precision, can be developed into a POC device for affordable analysis of RBC deformability for diagnostic applications (by measuring the sedimentation height of RBC samples during centrifugation). In this regard, different RBC samples with various rigidities were analyzed in the eLOD system, and their SI parameters were measured. The SI parameter and the response of the sensors for normal and rigid RBCs are shown in Fig. 4. In all experiments, the sensor array was set at a height of 14–18 mm. This placement was established during preliminary tests because, at this height, we observed a slower movement of the RBC-plasma interface. It can be seen that increasing the concentration of glutaraldehyde (GA) used to treat RBCs results in more rigid cells, leading to lower sedimentation indices. This process was captured in our recorded videos and used to evaluate the performance of the sensors. For instance, normal (healthy) RBCs have a steady SI parameter of 0.68, while hardened RBCs with 0.01% and 0.02% GA exhibit a steady SI parameter of 0.64 and 0.5, respectively (Fig. 4d). The difference in sedimentation index of different GA-treated samples is discernible even at the early stages of centrifugation (within 15 min). Accordingly, after 15 min of processing, the sensor signals can differentiate the GA-treated samples: two activated sensors (green circles) for normal RBCs, one activated sensor for 0.01% GA-treated RBCs, and no activated sensors for 0.02% GA-treated RBCs. The red-designated sensors indicate the placement of the RBC sedimented fraction over the corresponding sensor (which hinders the light rays and reduces the detected light intensity). Microscope images of normal and hardened RBCs are shown in Fig. 4e, f for further visual inspection. No specific variation in their shape was observed, which is consistent with other studies that highlight GA’s role in stiffening the cell membrane ^41^.

**Fig. 5:**
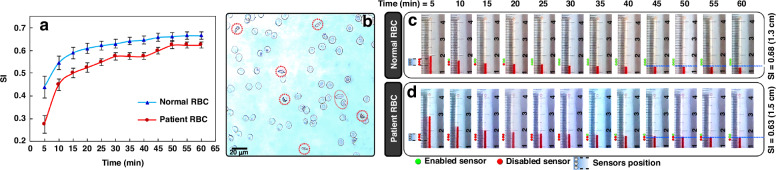
The photos of the fluidic channel, including healthy and patient blood samples during centrifugation, the response of the sensors, and the microscope photographs of blood samples. Measuring sedimentation height and ultimate sedimentation index (SI) for healthy and patient RBC samples at various time steps: **a** The graph of sedimentation index comparison between normal and patient RBC samples, based on video analysis to evaluate sensor performance. It shows average sedimentation values, with error bars representing standard deviations. **b** Microscope image of RBCs from a patient with sickle cell anemia, highlighting boat-shaped and sickle cells with red dotted circles as markers of the disease. **c** Sedimentation height was measured at 5-min intervals for normal and **d** patient-derived RBCs. Photographs of the channel and sedimentation progress were taken under synchronized strobe light for visual inspection. In all experiments, the sensor array was positioned at a height between 14 and 18 mm. Green circles indicate enabled sensors, while red circles denote disabled sensors, which are covered by sedimented RBC fraction, generating a shadow over the photosensor and reducing the detected light intensity. After 10 min of centrifugation at 1600 rpm, the number of enabled sensors revealed differences in RBC deformability, suggesting a possible diagnosis of sickle cell anemia (one enabled sensor for healthy RBC samples and no enabled sensors for patient RBC samples)

Similarly, the sedimentation index for patients’ RBCs was measured with this sensing mechanism, and the results are shown in Fig. 5a compared to healthy RBCs. The SI values of 0.68 and 0.63 were measured using the photography method for healthy and patient RBCs, respectively. Figure 5b shows a microscope image of RBCs from a patient with sickle cell anemia, highlighting various atypical cells, such as boat-shaped and sickle-shaped RBCs, indicated by red circles. The presence of these cells is a characteristic feature of sickle cell anemia^42^. We utilized these images to verify that the RBC samples from our patients are indeed affected by sickle cell anemia. Thus, RBCs obtained from patients with sickle cell anemia show decreased deformability (in bulk volumes) and settle at greater sedimentation heights, which can be used as diagnostic markers for the disease^44^. Consequently, the number of active sensors during centrifugation differs based on the sedimentation column height, with no sensors enabled for patient RBCs and one enabled sensor for healthy cells after 10 min (Fig. 5c, d). The significance of this method for analyzing RBC deformability is its simplicity and cost-effectiveness. Typically, measuring the deformability of individual RBCs cannot be done with simple light microscopes alone^45^ and usually requires additional laboratory pieces of equipment^46^. Our device provides a straightforward and affordable solution, facilitating the development of automated LOC platforms for analyzing various RBC samples and assisting in the diagnosis of related diseases ^38–40^.

### Numerical simulation

**Fig. 6:**
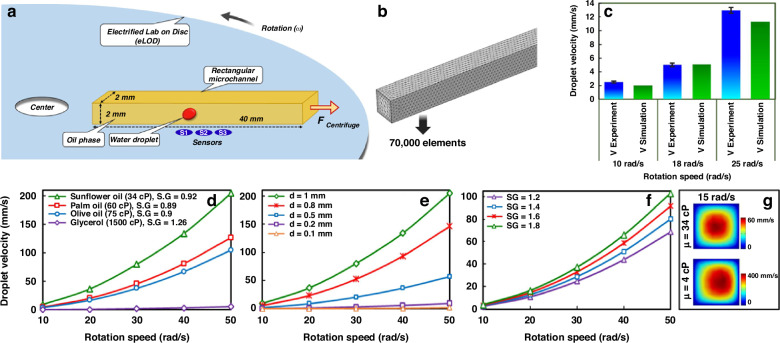
The simulation study results for predicting the movement of different droplets within a centrifugal channel. **a** Schematic illustration of the eLOD device, including the sensor array and channel dimensions. **b** The mesh structure of the numerical model was solved using the finite element method, consisting of 70,000 elements. **c** Validation of numerical simulations compared to experimental results for droplet velocity (*µ* = 75 cP, *d* = 1 mm, droplet specific gravity (SG) = 1). Graphs depict water droplet velocity at different rotation speeds for **d** various viscosities of the oil phase (*d* = 1 mm, SG = 1), **e** different droplet diameters (viscosity of the oil phase: *µ* =75 cP, SG of droplet = 1), and **f** different droplet densities (SG) (droplet diameter: *d* = 1 mm, *µ* = 75 cP). **g** Comparison of velocity magnitude for two different oil phases at the cross-section located at the midpoint of the microchannel (upper contour: *µ* = 34 cP, SG = 0.92, bottom contour: *µ* = 4 cP, SG = 0.85)

A computational model was utilized to predict the velocity of a water droplet within an oil phase at elevated rotation speeds, using the geometry illustrated in Fig. 6a and b. The model, constructed on a three-dimensional basis, accounted for different rotation speeds and various continuous phases in a counterclockwise rotation mode. The validation of the numerical simulations against experimental findings demonstrated good agreement and reliability (Fig. 6c). Various oil phases with different viscosities (µ), such as sunflower, palm, olive, and glycerol oils, were examined (Fig. 6d). As the viscosity of the oil phase increases, the velocity of the water droplet decreases. For instance, with the highest viscosity, glycerol oil results in the slowest velocity, even at high rotation speeds. Moreover, the size (diameter) of the droplets (d) significantly influences their velocity, with larger droplets moving faster than smaller ones (Fig. 6e). Similarly, higher-density droplets tend to move at a greater velocity (Fig. 6f). Generally, at higher rotation speeds of the eLOD, droplets attain higher velocities due to the predominance of centrifugal force. Figure 6g shows the velocity magnitude at a cross-section of the microchannel for two viscosity values. The Coriolis force clearly pushes the fluid with lower viscosity (~4 cP) towards the right-side wall of the microchannel, while the more viscous fluid exhibits no such deviation.

## Conclusions

In this study, we introduced an optical sensing mechanism using an array of LDR sensors with cone-shaped waveguides, enabling detection at sub-millimeter resolution in microfluidics. We chose to use LDRs because they offer accurate light intensity measurements while being cost-effective than other sensors (e.g., ten times cheaper than RGB sensors). We applied this sensing mechanism to an eLOD device that offers wireless connectivity to smartphones or laptops for extended processing capabilities. The study emphasizes the critical role of system operating parameters in determining light intensity when using waveguide converters. Key factors include LED power, microchannel depth, the number and diameter of apertures, the gap between them, and the tilting angle of the cones. The system demonstrated sensitivity for microchannels depths as low as 80 µm, with greater depths increasing light absorbance and enhancing the differentiation of colorful fluids. Apertures can be customized in number and spacing to suit specific applications, with a minimum gap of 0.3 mm determined by fabrication limitations. The tilting angle significantly affects LI, with lower angles preferred due to reduced distances between the apertures and the LDR. Aperture diameter, influenced by the microchannel width, particle or droplet size, and boundary movement, enables sensitivity to feature as small as 200 µm. Our proposed sensing mechanism has various applications, such as detection and velocity measurement of droplets/particles, sample volume monitoring, analyte concentration analysis, and RBC deformability analysis in bulk form. These applications are enabled by detecting light intensity variation in fluid media, exploiting the ability of linear arrays of photosensors. Moreover, these versatile sensors have potential diagnostic and chemical engineering applications. We conducted experiments to understand the sensors’ responses concerning color concentration, aperture diameter, and droplet velocity. Additionally, we employed an array of three sensors to assess the deformability of RBCs by measuring the sedimentation height in their bulk form. Notably, our method successfully distinguished RBCs from individuals with sickle cell anemia compared to healthy RBCs, indicating its potential as an automated diagnostic tool in the future.

Numerical simulations revealed the significant influence of the viscosity of the continuous phase on droplet velocity at high rotation speeds. Given the high potential for automation in centrifugal platforms and the established unit operations on these platforms (e.g., various passive and active valves, serial dilution, washing, cell separation, etc.), this sensing mechanism can be beneficial for future sample-to-answer detection devices, particularly in extreme point-of-care settings and for diagnostics of conditions such as thalassemia, diabetes, and malaria. Researchers can customize the number and position of the sensors as needed. Our research team is working on an enhanced version of this sensing method, incorporating a more compact eLOD using induction power transmission, a wider field of view with higher resolution, and incorporation with additional unit operations.

## Methods

For fabricating the proposed eLOD device, we used the following electronic and mechanical parts: Arduino UNO as a microcontroller (Smart Projects, Milan, Italy), Bluetooth module for transmitting signals (OEM, 2.4 GHz), 9 V battery pack (providing ~4 h of continuous operation and longer for intermittent use), LEDs (white color, diameter = 5 mm, 1 W), LDR sensors (GL 5506, OEM, China), servo motor (100 W, 3000 rpm, Delta Electronics, Taipei, Taiwan), and servo drive (Delta Electronics, Taipei, Taiwan). A stroboscopic light source (Movistrob 2000, BBB, Arnsberg, Germany) was used to capture images of sedimentation height during eLOD centrifugation to validate the sensors’ performance. Additionally, a wireless digital microscope (OEM) was positioned above the fluidic channel to record videos of droplet movements for further validation of the sensors’ performance. Furthermore, different dyes (water-soluble, food-grade yellow, pink, and blue from Enco, Mexico) and oil-based paints (painting pack from Rodin, Mexico) were used to prepare colorful liquids for sensor-assessment experiments. Aqueous dye solutions (2.5% in distilled water) and oil-based solutions (5% in transparent mineral oil) were initially prepared in primary colors and designated as 100% stock solutions. Additional colors and concentrations were then created by diluting or mixing the stock solutions in water or oil to achieve 100%, 50%, 25%, and 12.5% concentrations (Fig. 2c, d). The resulting colorful liquids were transferred into the wells of a 24-well plate, with 500 µL in each well. The LI values of each liquid were measured using a sensor with a 1 mm aperture size (at 3 V power of LED). An optical microscope (SZX7, Olympus, Tokyo, Japan) was used to photograph RBC morphology.

### Device preparation

The fluidic channels were fabricated using a CO_2_ laser cutter machine (100 W, CMA-1390 Yueming laser, Dongguan, China) on transparent sheets of polymethyl methacrylate (PMMA) and polyvinyl chloride (PVC, thickness = 100 µm, GBC) and double-sided pressure sensitive adhesive (PSA, thickness, =80 µm, Flexcon, Massachusetts, USA). Standard methods were used for preparing the fluidic chips, which involved bonding the top and bottom PMMA layers to the middle PVC layer (on which the channel was cut) using double-sided pressure-sensitive adhesives^47^. The primary channel dimensions were 40 × 2 × 2 mm³ (L × W × H), including the inlet and outlet holes. For the microchannel depth experiments, various depths were achieved by stacking layers of PVC and PSA, resulting in channel depths of 80, 260, 460, 620, and 800 µm.

For diagnostic experiments, the width of the fluidic channel is considered to be 1 mm, providing an 80 µL capacity for blood samples. Various sub-millimeter depths of the channel were also examined in the sensor calibration section. This device consists of two parts: (1) the fluidic chip and (2) the electronic compartment (Fig. 1c). The electronic compartment houses a microcontroller, Bluetooth^®^ module, battery pack, and sensor arrays (cone-modified LDR photodetectors). This compartment is positioned beneath the fluidic channels on the rotating platform (Fig. 1d, e) and balanced equally at two sides for vibration-free movement during rotation. The 3D-printed cone-shaped waveguides (Fig. 1b represents the photos, sketch, and dimensions of the waveguides) were placed over the LDRs, and the cones’ internal walls were covered with reflective aluminum coating to ensure each cone was light-proof and transmitted light only from the top aperture. Additionally, they were filled with translucent epoxy resin (KER 828 and polyamine) to enhance illumination guidance. The gap between the cones can be customized based on the application’s requirements, with the minimum value constrained by fabrication precision. LEDs were installed directly above the fluidic channel, opposite the sensor arrays, at a distance of 1 cm. The waveguide can be fabricated with two to six apertures, covering distances ranging from 2 mm to 1 cm. Additionally, multiple waveguides (i.e., LDRs) can be connected to a microcontroller using a multiplexer, allowing for the monitoring of longer lengths if needed. Furthermore, waveguide structures can be positioned side-by-side along the length of the microchannel. These configurations facilitate process monitoring over extended lengths, effectively overcoming the limitation of a short field of view. The 3D illustration of waveguides with multiple cones (featuring two to six apertures) is presented in Fig. 1f, highlighting their potential for extended use in various applications.

### Preparation of RBC samples

**Fig. 7:**
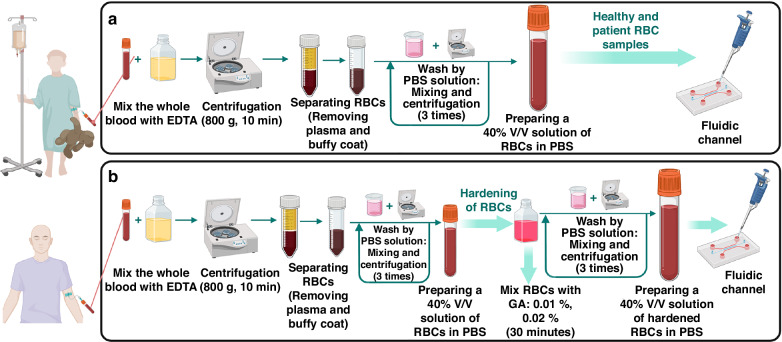
Illustrations of the different steps involved in preparing blood samples before experiments. Preparation process of blood samples for use in the eLOD device: **a** Collecting and preparing RBCs from healthy and patient donors. **b** Using GA to reduce the deformability of healthy RBCs

Human whole blood samples were obtained from Isfahan University of Medical Science following safety regulations and approved collection methods by the ethics committee. The samples were drawn with anticoagulant (EDTA, Ethylenediaminetetraacetic acid), stored at 4 °C, and analyzed within one hour. RBCs were separated by centrifugation, followed by removal of the buffy coat and plasma, and then washed three times with phosphate-buffered saline (PBS). Samples containing 40% V/V of RBCs were prepared by resuspending them in PBS. Normal RBCs were treated with 0.01% and 0.02% GA at 25 °C for 30 min to increase rigidity, a common method in RBC deformability studies. After the hardening process, RBCs were rewashed and dispersed in PBS at 40% V/V. Similarly, RBCs from sickle cell anemia patients were washed and prepared for analysis on the eLOD device. All RBC samples were prepared at an identical concentration (40% V/V in PBS solution), enabling the comparison and measurement of cell deformability. Figure 7 represents the procedure for preparing blood samples used in the sedimentation experiments.

### Experiments

The experiments to evaluate the performance of optical sensors were categorized as follows:

#### LDR calibration

Light absorption measurements were conducted under various conditions to calibrate the sensors. This included assessing color variations by measuring the light intensity passed through dyed fluids at different dye concentrations, sensing the interface of a two-phase fluid, and counting the number of water-in-oil droplets in the fluidic channel. Similar calibration techniques can be applied to use this sensor array to detect the color-dependent concentrations of specific analytes.

#### Water/oil droplet study

The speed of dyed water droplets traveling within an oil continuous phase in the fluidic channel was measured at various rotation speeds. The response time of the sensor array was recorded and compared with video captures taken using an on-disc wireless microscope. Droplets were manually generated using syringe needles of various sizes and introduced into the device to analyze their movement and impact on the sensors. Depending on specific application requirements, various methods for droplet generation in centrifugal microfluidics can be utilized^48^. Images and videos of the droplets and fluid boundaries during rotation were captured using a wireless microscope (SV606, SVBONY, Kowloon, Hong Kong).

#### Biomedical study

The RBC sedimentation time and index were assessed at a rotational speed of 1600 rpm. This was done using an array of photosensors and synchronized strobe lighting for image capturing to evaluate the sensors’ performance. This analysis was performed on (I) a sample of healthy RBCs, (II) hardened RBCs treated with different concentrations of GA (0.01% and 0.02%) to assess deformability, and (III) RBC samples from patients with sickle cell anemia disease.

The rotation of the eLOD device was facilitated by a servo motor controlled via a servo drive connected to PC software. During rotation, the signals of light absorbance from sensors were wirelessly transmitted to the Arduino IDE 2.1.0 software via Bluetooth^®^ for further analysis (Fig. 1d). The deformability of RBCs was quantified using the SI parameter, represented by Eq. 1. Here, *d*_0_ (=41 mm) and d_1_ denote the channel length and sedimentation height at various time intervals, respectively (see Fig. 1e). The SI parameter was monitored and recorded under synchronized stroboscope lighting, and the results were compared with the sensors’ signals.

1 $${\rm{SI}}=\frac{{{\rm{d}}}_{0}-{{\rm{d}}}_{1}}{{{\rm{d}}}_{0}}$$

In this study, all experiments were repeated ten times (*N* = 10), and the results are presented as the average values ± standard deviation.

### Numerical study

We conducted a 3D numerical study employing finite element analysis (COMSOL Multiphysics, V 6.0) to expand upon the water/oil droplet experimental investigation. This study encompassed a broader spectrum of rotational speeds and diverse liquid phases. Here, we calculated the velocity of water droplets across various liquid phases and rotational speeds, validating our findings with experimental data. Detailed theoretical background and governing equations are provided in the electronic supplementary information (ESI).

## Supplementary information

Supplementary document

Video S1

Video S2
