# Supplementary material for: Low-cost optical sensors in electrified lab-on-a-disc platforms: liquid-phase boundary detection and automated diagnostics
Source: Microsyst Nanoeng. 2025 Apr 7;11:61. doi: 10.1038/s41378-025-00896-5 (PMC11977271; doi:10.1038/s41378-025-00896-5)
Supplement: Supplementary file 1 — Supplementary document [file 41378_2025_896_MOESM1_ESM.docx]

**Low-cost Optical Sensors in Electrified Lab-on-a-Disc Platforms: Liquid-Phase Boundary Detection and Automated Diagnostics**

Vahid Kordzadeh-Kermani^1,2^, Maryam Vahid^3^, Seyed Nezameddin Ashrafizadeh^2^, Sergio O Martinez-Chapa^1^, Marc J Madou^*1,4^, and Masoud Madadelahi*^1^

^1^ School of Engineering and Sciences, Tecnologico de Monterrey, Monterrey, 64849, NL, Mexico.

^2^ Research Lab for Advanced Separation Processes, Department of Chemical Engineering, Iran University of Science and Technology, Narmak, Tehran 16846-13114, Iran.

^3^ Department of Mechanical Engineering, Isfahan University of Technology, Isfahan, 84156-83111, Iran.

^4^ Department of Mechanical and Aerospace Engineering, University of California Irvine, Irvine, CA, 92697, USA


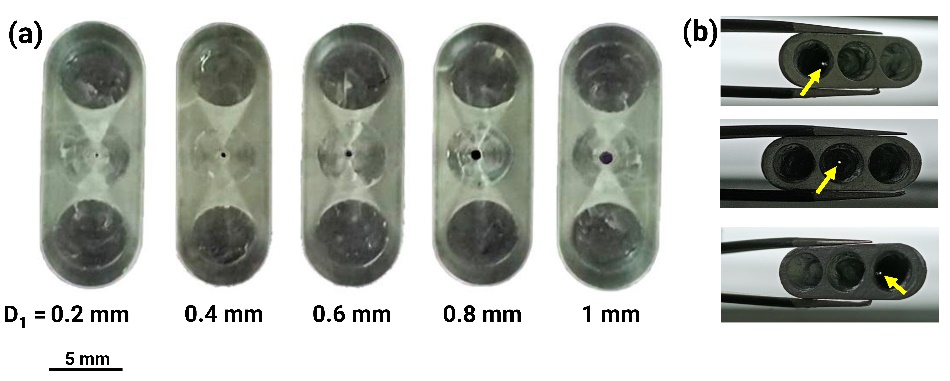


**Fig. S1.** (a) Photograph of waveguides with triple apertures of varying diameters (0.2, 0.4, 0.6, 0.8, and 1 mm) before treatment for experimental use. (b) Photographs of a waveguide with 200 µm aperture diameters from different angles after light-proofing treatment. The yellow arrows indicate the 200 µm apertures transmitting light within the cone.


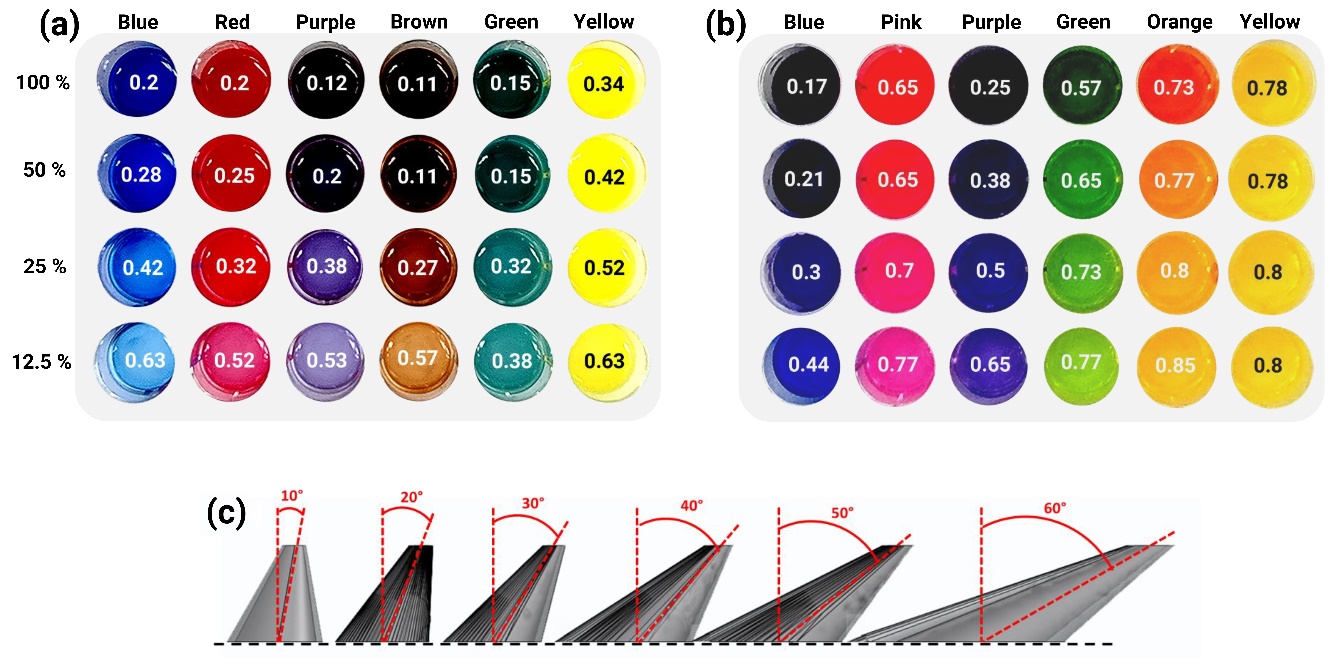


**Fig. S2**. (a) Light Intensity (LI) measurements for various colorful liquids at different concentrations in a 24-well clear plate using oil-based dyes (liquid volume = 500 ml, LED power = 3 V). The numbers on the left-hand side indicate dye concentrations, while the numbers inside the circles represent the corresponding LI values. (b) Graphs showing LI measurements for water-based dyes under similar conditions. Results are presented as averages with a 5% deviation. (c) Illustration of the waveguide at various tilting angles, ranging from 10° to 60°.


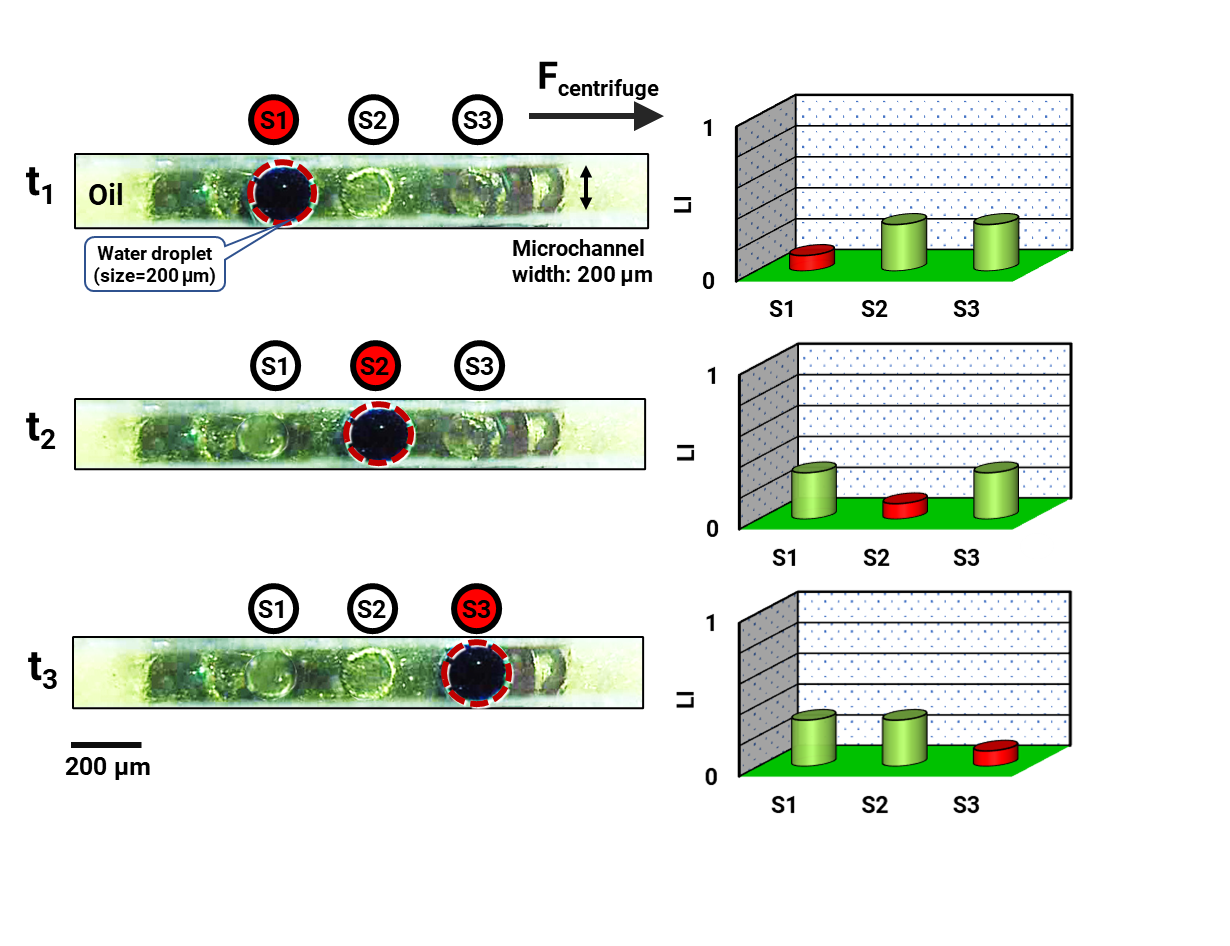


**Fig. S3.** Photographs of a 200 µm diameter dyed water droplet in continuous mineral oil flowing over triple sensors, each with a 200 µm aperture size. The width of the microchannel (yellowish color) is 200 µm, and the droplet velocity is 1 mm/s. The images were captured using a wireless microscope mounted on the rotating platform, and the size of the components, including the droplet diameter, was measured using the same microscope.

**Governing equation for centrifugal microfluidics**

The governing equations, which exploits in the numerical studies of fluid flow and particle tracing in centrifugal, are presented here and elaborated in the reference^1^.

The amount of flow in the rotating microchannel is determined by solving continuity and Stokes equations by considering centrifugal forces (Equations 1 and 2, $\vec{u}$: velocity vector, $\rho_{f}$: fluid density, $P$: pressure, $\mu_{f}$: dynamic viscosity, $\vec{f}$: body forces summation). In the rotating microfluidic platforms, the body forces exerted on the volume of fluid can be considered as centrifuge and Coriolis forces (Equations 3, 4, and 5, $\vec{r}$: radial distance from the center of rotation, $\vec{\omega}$: vector for angular velocity).

$\nabla u=0$ (1)

$\rho_{f}\left( \partial_{t}+\vec{u}.\nabla\right)\vec{u}=-\nabla P+\mu_{f}\nabla^{2}\vec{u}+\vec{f}$ (2)

$\vec{f}=\vec{f}_{Centrifugal}+\vec{f}_{Coriolis}+\vec{f}_{Lift}$ (3)

$\vec{f}_{Centrifugal}=-\rho_{f}\vec{\omega}\times\left( \vec{\omega}\times\vec{r} \right)$ (4)

$\vec{f}_{Coriolis}=-{2\rho}_{f}\vec{\omega}\times\vec{u}$ (5)

Furthermore, to track particles within the steady-state, rotating microfluidic platform, Newton’s laws, encompassing Centrifugal, Coriolis, Drag and Lift (Saffman) forces were applied, as described in Equations 6 to 12 ($m_{d}$: droplet mass, $v_{d}$: droplet volume, $\rho_{d}$: droplet density ,$v_{t}$: relative velocity of fluid and particle, $d$: droplet’s diameter, $\gamma$: shear rate, $\vec{V}_{f}$: fluid velocity vector, $\vec{V}_{P}$: droplet velocity vector, $k$: Saffman constant ). In this context, forces like Brownian are negligible on particles because the primary forces (*i.e.,* Centrifugal and Coriolis) exert such a dominant influence that no discernible differences are observed during processing.

$\frac{d\left( m_{d}v_{d} \right)}{dt}=F_{total}=F_{Centrifugl}+F_{Coriolis}+F_{Drag}+F_{Lift}$ (6)

$F_{Centrifugl}=-\left( \rho_{d}-\rho_{f} \right)\frac{\pi d^{3}}{6}\vec{\omega}\times\left( \vec{\omega}\times\vec{r} \right)$ (7)

$F_{Coriolis}=-\left( \rho_{d}-\rho_{f} \right)\frac{\pi d^{3}}{6}\vec{\omega}\times\vec{V}_{d}$ (10)

$F_{Drag}=3\pi\mu_{f}dv_{t}$ (11)

$\vec{F}_{Saffman}=kd^{2}\left( \vec{V}_{f}-\vec{V}_{d} \right)\left( \mu_{f}\rho_{f}\gamma\right)^{\frac{1}{2}}$ (12)

**Reference**

1 Madadelahi M, Acosta-Soto LF, Hosseini S, Martinez-Chapa SO, Madou MJ. Mathematical modeling and computational analysis of centrifugal microfluidic platforms: a review. *Lab Chip* 2020; **20**: 1318–1357.
